# Airborne Infection with *Bacillus anthracis*—from Mills to Mail

**DOI:** 10.3201/eid1006.020738

**Published:** 2004-06

**Authors:** Kevin P. Fennelly, Amy L. Davidow, Shelly L. Miller, Nancy Connell, Jerrold J. Ellner

**Affiliations:** * New Jersey Medical School–UMDNJ, Newark, New Jersey, USA;; † University of Colorado, Boulder, Colorado, USA

**Keywords:** Anthrax, Air microbiology, Infection, Risk, Inhalation exposure, Lethal Dose 50, Ventilation

## Abstract

The lack of identified exposures in 2 of the 11 cases of bioterrorism-related inhalation anthrax in 2001 raised uncertainty about the infectious dose and transmission of *Bacillus anthracis*. We used the Wells-Riley mathematical model of airborne infection to estimate 1) the exposure concentrations in postal facilities where cases of inhalation anthrax occurred and 2) the risk for infection in various hypothetical scenarios of exposure to *B. anthracis* aerosolized from contaminated mail in residential settings. These models suggest that a small number of cases of inhalation anthrax can be expected when large numbers of persons are exposed to low concentrations of *B. anthracis*. The risk for inhalation anthrax is determined not only by bacillary virulence factors but also by infectious aerosol production and removal rates and by host factors.

The intentional release of *Bacillus anthracis* spores through the mail in the United States in the fall of 2001 was associated with inhalation anthrax in 11 persons, 5 (45%) of whom died (1,2). Seven cases were associated with occupational exposures in the postal service, and two case-patients had documented exposures to contaminated mail in the business office of a media company. No sources of exposure were identified for two women who were presumably exposed to secondarily contaminated mail.

*B. anthracis* was previously known to have potential as a weapon on battlefields or for large-scale outdoor dissemination. Its delivery through the mail moved the risk indoors. We sought to improve our knowledge of indoor transmission of *B. anthracis* by applying principles and methods used in studies of tuberculosis. Although room ventilation and other environmental factors are known to be important in the airborne transmission of *Mycobacterium tuberculosis*, little attention has been given to these factors in the transmission of *B. anthracis*. We used a mathematical model of airborne infection to theoretically assess risk for infection with *B. anthracis* from indoor exposures; to demonstrate the relative importance of pathogen, environmental, and host factors in transmission; and to estimate a range of infectious doses on the basis of these models and the epidemiology of the 2001 anthrax cases.

## Methods

We reviewed the literature in English on inhalation anthrax by searching MEDLINE electronically and by obtaining articles cited in references in these and older articles. We focused on occupational cases and outbreaks and on experimental aerosol exposures of nonhuman primates that included environmental sampling data. We used the Wells-Riley modification of the Soper and Reed-Frost models of infection as our mathematical model of airborne infection (*3**–**5*). This equation can be arranged so that the probability of airborne infection is given by *P (infect) = C/S=1-exp(-Iqtp/Q)*, where C is the number of cases among S persons susceptible to the infection; I is the number of sources of infection; q is the number of "quanta," or units of bacilli necessary to cause infection produced per source per unit of time; t is the time of exposure per unit of time; p is the minute ventilation rate of the exposed susceptible hosts in volume per unit of time; and Q is the volumetric rate of fresh air ventilation that removes the infectious aerosol in volume per unit of time. Units used were cubic feet and minutes. Biologically, a quantum may be one or more spores in one or more airborne particles, analogous to a colony-forming unit on a culture plate. Statistically, a quantum is the amount of infectious material needed to produce infection in 63% of uniformly exposed animals, or 1.25 times the median infectious dose, i.e., q = 1.25 x ID_50_ (*5*). We modeled transmission of infection from contaminated mail in two general environments: 1) the workplace of mail distribution centers and 2) home or office exposures.

Using published attack rates of inhalation anthrax of 0.25% (2/750) and 0.66% (4/610) in two U.S. Postal Service (USPS) processing and distribution centers in New Jersey (*6*) and Washington, D.C (*7*), respectively, we estimated the quanta produced for a range of available workplace ventilation rates by using the Wells-Riley equation above and solving for q. Data on the size and ventilation of the processing and distribution center workrooms were obtained from a USPS ventilation engineering contractor (C. Hong, pers. comm.). For the exposures in the USPS postal distribution centers, we assumed an exposure time of 8 hours to represent a work shift and a pulmonary ventilation rate of 14.6 L/min (0.5156 ft^3^/min), as might be expected with moderate work. This rate is comparable to the rate used to estimate inhaled doses in a study of an anthrax outbreak that occurred in a goat hair–processing mill in Manchester, New Hampshire, in 1957 (*8*).

We also estimated the risks for airborne infection associated with hypothetical exposures to *B. anthracis* that might have occurred during the handling and opening of contaminated mail in a home or office. For the first of these modeling scenarios, we used room dimensions of 12 ft x 18 ft x 8 ft (3.7 m x 5.5 m x 2.4 m), or a volume of 2,160 ft^3^ (61 m^3^), which is a reasonable size for an office or room in a home and allows for comparison to modeling data on occupational tuberculosis. We then modeled the risks for exposure in a smaller room of 56 ft^2^ x 8 ft high (432 ft^3^, 12.2 m^3^) and for exposure as a result of dispersion throughout a median-sized U.S. house, which has a volume of 10,947.5 ft^3^ (310 m^3^) (*9*).

We modeled room ventilation rates of 0.5 and 2 air changes per hour that correspond to 18 ft^3^ and 72 ft^3^ (0.5 m^3^ and 2.0 m^3^) per minute. This rate approximates a current standard for fresh air ventilation of 20 ft^3^/min (0.57 m^3^/min) per occupant for office space and 15 ft^3^/min (0.42 m^3^/min) per person for residential living areas, if one assumes that only one person occupied the room (*10*). We assumed duration of exposure of 1 hour to simulate an exposure occurring with the opening of contaminated mail with gradual dispersion of the aerosol. We included pulmonary ventilation rates of 4 L/min, 6 L/min, and 10 L/min for exposed persons to span the range of minute-ventilation reported in the literature for persons at rest (*11**–**13*).

Because our review of the literature did not provide a conclusive infectious dose for humans, we followed the approach first suggested by Wells (*4**,**5*) of using units of quanta to represent the unit required to establish infection, as has been used in studies of tuberculosis and measles (*4**,**14*). Since the infectious dose of inhaled virulent anthrax spores cannot ethically be determined experimentally in humans, we estimated the 50% infectious dose (ID_50_) based on data from the 8-hour inhaled dose measured in Manchester in 1957. The inhalation dose for workers in the area where four of the five cases of inhalation anthrax had occurred was estimated to be 140–690 spores <5 µm in size (and 620–2,200 total spores), based on air sampling data (*8*). Sixteen presumably susceptible workers were in this area (*15*), so this estimate may be considered an ID_25_, since 25% of the exposed persons were infected. On the basis of studies of 1,236 monkeys, Glassman estimated the median lethal dose to be 4,130 spores, and using the probit model he suggested that the LD_25_ was associated with a 10-fold decrease in dose (*16*). When this principle is applied, a 10-fold increase of the ID_25_ for the Manchester mill outbreak would result in an ID_50_ of 1,400 to 6,900 spores <5 µm, or 6,200–22,000 total spores (*8*). This number is similar to Glassman’s estimate and to the lower range of the LD_50_, 2,500–55,000, accepted by an expert panel (*17*). Since q = 1.25 x ID_50_, then one quanta under these assumptions is 1,750–8,625 spores <5 µm (7,750–27,500 total spores).

The number of persons exposed to *B. anthracis* spores through the mail in the fall of 2001 is unknown, so we arbitrarily expressed the probability for infection as the number infected per 10,000 population. Estimates of the numbers of persons infected can be calculated by using these estimates. For example, if approximately 5,000 persons were exposed to contaminated letters as might be suggested by the modeling data of Webb and Blaser, these risks can be divided by two. All data were entered, stored, and analyzed by using JMP-SAS software (version 4.0.4, SAS Institute, Cary, NC.)

## Results

Inhalation anthrax developed in two of the 750 persons working in the USPS processing and distribution centers in Trenton, New Jersey, developed , for an overall attack rate of 0.25% (*6*). The workroom is 151,200 ft^2^, with a height of 15 ft, for a total volume of 2,268,000 ft^3^ (C. Hong, pers. comm.). The area is ventilated with 5.29 air changes per hour, with a minimum of 11.8% and a maximum of 100% of fresh air. Therefore, the nonrecirculated air-ventilation rate varies from a minimum of 23,596 ft^3^/min to a maximum of 199,962 ft^3^/min. Solving for q results in an exposure of 0.238 to 2.08 infectious quanta per minute, or 114 to 998 infectious quanta generated per 8-hour shift. If one assumes that one quantum is 1,750–8,625 spores <5 µm (7,750–27,500 total spores), then the 8-hour cumulative production of infectious aerosol is estimated to have ranged from 199,500 to 8,607,750 spores <5 µm (883,500–27,445,000 total spores).

Similar calculations were performed for the processing and distribution center in Washington, D.C., where inhalation anthrax developed in 4 of 610 workers (attack rate 0.66%). The workroom is 4,605,000 ft^3^ (C. Hong, pers. comm.) and is ventilated at 2.88 air changes per hour, with a minimum of 16.2% outside air. The calculated ventilation rate is 35,809–221,040 ft^3^/min, resulting in a range of q of 0.96–6.0 infectious units per minute, or 461–2,880 quanta per 8-hour shift. When the same assumptions of the number of bacilli per quantum are used, the 8-hour cumulative production of infectious aerosol is 806,750–24,840,000 spores <5 µm (3,572,750–79,200,000 total spores).

The risks for airborne infection to susceptible occupants in our hypothetical scenarios of exposure in a home or office are summarized in the Table and plotted in the Figure. The risks for infection associated with exposure to one quantum in these models is 6–936 per 10,000 persons. If the exposure concentration decreases by one log, i.e., to 0.1 quantum, the risk decreases to 0.6–98 per 10,000 persons. At exposure concentrations >0.1 quantum, the risks are >6 cases per 10,000 and increase to very large magnitudes.

**Table:** Risk for airborne infection with *Bacillus anthracis* modeled for various scenarios of exposure to secondarily contaminated mail for 1 hour in a home or office, expressed as number of cases per 10,000 susceptible persons exposed^a^

Quanta	Small room	Moderate-size room						House
	0.5 ACH	0.5 ACH			2 ACH			2 ACH
	Pulmonary ventilation							
	10 L/min	10 L/min	6 L/min	4 L/min	10 L/min	6 L/min	4 L/min	4 L/min
0.001	1.0	0.2	0.1	0.1	0.05	0.03	0.02	0.006
0.01	9.8	2.0	1.2	0.8	0.5	0.3	0.2	0.1
0.1	97.8	19.6	11.8	7.9	4.9	3.0	2.0	0.6
1	936	195	117	78	49	30	20	6
10	6,256	1,784	1,113	756	479	291	195	61
100	10,000	8,599	6,928	5,443	3,881	2,555	1,784	596
1,000	10,000	10,000	9,999	9,996	9,926	9,477	8,598	4,588

^a^ACH, air changes per hour.

**Figure:**
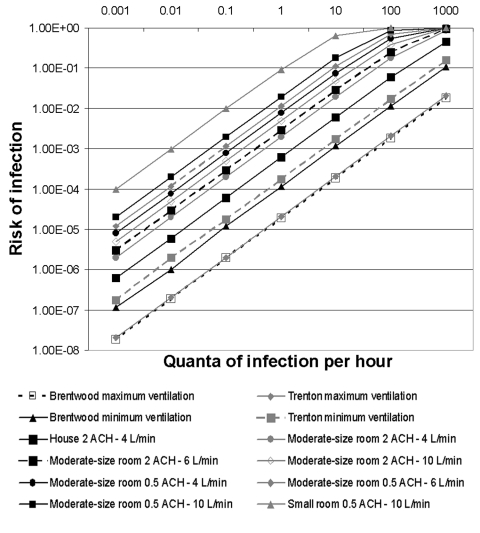
Risk for airborne infection with *Bacillus anthracis* in various scenarios. Home and office exposures are for 1 hour, and postal facility exposures are for 8 hours; for postal facilities, the models assume a 14.6 L/min pulmonary ventilation rate with moderate work, comparable to the rate used to estimate inhaled doses in the Manchester study. ACH, air changes per hour.

If 1 quantum is 1,750–8,625 spores <5 µm, then inhaled doses corresponding to lower exposures can be calculated: 0.1 quantum would be 175–863 bacilli, 0.01 quantum would be 18–86 bacilli, and 0.001 quantum would be 2–9 bacilli. Exposures estimates <0.001 quantum are biologically meaningless under these assumptions since fractions of spores are not viable.

Changes in room size and ventilation rate affect risk more than changes in pulmonary ventilation in these scenarios. The relative risk reduction associated with an increase of room ventilation from 0.5 to 2 air changes per hour is highest for low-concentration exposures and decreases with increasing concentration of the aerosol exposure. For example, in the scenario of a high-concentration exposure of 100 quanta and the exposed person’s breathing 10 L/min in the moderate-size room with 0.5 air changes per hour, improving ventilation to 2.0 air changes per hour decreases the risk by 55%. However, if the same person is exposed to 0.1 quantum in the same room with the same changes in ventilation, the risk decreases by 75%. As expected, for a given exposure concentration and pulmonary ventilation rate, the lowest risk is in the large, well-ventilated space and the highest risk is in the small, poorly ventilated room. For example, if exposure is 1 quantum of infection to a person breathing 6 L/min, the risk in the small, poorly ventilated room is 58.9 per 10,000 and in the large, well-ventilated space, the risk is 0.6 per 10,000.

The Figure illustrates that, although the production of infectious aerosol and the duration of exposure were higher for postal workers in the postal distribution center facilities, the greater rate of removal of the infectious aerosol by dilution ventilation in such facilities results in a lower risk for infection for a given aerosol production rate (q) than in the home exposure scenarios.

## Discussion

These analyses emphasize that the risks for airborne infection are determined not only by the virulence of the agent but also by environmental factors, e.g., room size and ventilation rates, and host factors, e.g., pulmonary ventilation rate. Little can be done to change one’s pulmonary ventilation rate, but these data suggest that risks for inhalation anthrax are likely to decrease considerably with fairly modest increases in room ventilation for low-concentration exposures. These data might be helpful in educating both the general public and policymakers regarding strategies to reduce risks from aerosolized bioweapons.

General dilution ventilation reduces the risk of transmitting airborne pathogens; however, as described for *M. tuberculosis*, room ventilation is theoretically limited in decreasing this risk, especially when the exposure concentration is high (14). UV germicidal irradiation has been suggested as a potential bioterrorism countermeasure (*18*). Although we are not aware of data regarding the use of irradiation to inactivate *B. anthracis*, Peng and colleagues found that UV germicidal irradiation of upper-room air reduced *B. subtilis* spore concentrations by 46% to 80%, depending on the ventilation rate (*19*). Thus, further research on the use of UV germicidal irradiation and other interventions seems warranted.

These modeling data suggest that approximately 0.7–63.4 million total airborne spores and approximately 0.2–20 million spores <5 µm may have been produced by contaminated mail during work shifts at the two USPS processing and distribution centers where inhalation anthrax developed in postal workers. These data are plausible, given the contamination with *B. anthracis* documented by environmental sampling of both the New Jersey and the Washington, D.C., facilities (*6**,**20*). Although the results of air sampling were negative, dust samples grew 3–9.7 million CFU/g (*20*). A contaminated letter in Washington, D.C., purportedly contained 2 g powder with 100 billion to 1 trillion spores per gram (*21*), which suggests that letters can be a source of infectious material.

These data also suggest that a few cases of inhalation anthrax can be expected if large numbers of people are exposed to a virulent strain at low concentrations within homes or offices. We will probably never know how many persons were exposed to mail contaminated with *B. anthracis* in the fall of 2001. If 10,000 persons were exposed in a home or office to secondarily contaminated mail, and two persons were infected, these models suggest that exposure was probably to 0.1 or 0.01 quantum, or 18–863 airborne spores. Above and below that range of exposures, the number estimated to be infected is very large or very small. Even if 1,000 persons were exposed, this range of exposures appears most reasonable, except for exposures to 1 quantum in larger or well-ventilated environments.

Glassman suggested that 100 spores may be sufficient to cause infection (*16*), and a more recent analysis suggested that as few as 1–3 spores may be sufficient to cause infection (*22*). Our data are consistent with these conclusions, as 0.001 quantum would be 2 to 9 bacilli in our model.

We assumed in our analyses that the strain used in the mailings was at least as virulent as the strain reported in the outbreak of inhalation anthrax at the Manchester mill (*15*). Genomic analysis recently identified the isolate used in the mailings as the Ames strain, which is used in multiple research laboratories (*23*). However, the strain was prepared for aerosolization in powder form, and we are not aware of published data on infectious doses for powdered preparations. The method of preparing bacilli for aerosolization may affect retention of aerosolized agents and their virulence of *B. anthracis*. For example, the surface-active compound Tergitol was found to increase virulence 10-fold in guinea pigs (*24*).

The two women with no identified exposures in the recent bioterrorism-related outbreak may have been unusually susceptible to inhalation anthrax. Risk factors for increased susceptibility or resistance to inhalation anthrax are not known, although age has been suggested (*25*). One of these women was 61 years old (*2*), and the other was 94 years old (*1*). Aging is associated with a decrease in mucociliary clearance as well as alterations in immune responses, but given the large numbers of elderly persons who were likely exposed to contaminated mail, age alone seems inadequate to explain the epidemiology of this outbreak. We are reluctant to accept that children are less susceptible than elderly adults without additional data. Previous reports of cases associated with industrial sources or with materials contaminated by *B. anthracis* suggest that some susceptible persons, including children, were infected during relatively brief exposures (*26**–**28*). We speculate that the combination of thin body type, age, and female sex might be a risk factor for inhalation anthrax, as it is for pulmonary infections with environmental nontuberculous mycobacteria, e.g., with *M. avium* complex (*29*). Women with asymptomatic bronchial hyperresponsiveness have increased deposition of inhaled particles of ≈1 µm (*30*), but we are not aware of data that show this to be a risk factor for respiratory infections.

Modeling studies of transmission of tuberculosis or measles have usually assumed a single uniform pulmonary ventilation rate in exposed persons as well as a homogenous concentration of infectious aerosol over time and space. Riley’s choice of 10 L/min to model human ventilation has been used by other modelers (*14*), but we have added a broader range of values to assess the relative importance of variability in minute ventilation. However, human ventilation is not uniform, being punctuated by sighs in which the tidal volume may be three times greater than the volume at rest (*12*). If a piece of contaminated mail were opened, aerosol was likely concentrated immediately afterwards and in the immediate vicinity. Thus, if a person were to sigh when exposed to the maximum number of airborne spores, spores would likely deposit in greater concentration. However, we are not aware of data documenting deposition associated with short-term irregularities in ventilation. This is another area for further research.

Druett first applied the probit model in early studies of inhalation anthrax in animal models (*31*). He acknowledged that this model best fit the data within the range of the LD_25_–LD_75_ but cautioned about interpreting data outside that range. Our concern with estimating the number of bacilli required to kill roughly 0.1%–1.0% of exposed persons (10–100/10,000) was one of the reasons we chose not to apply the Druett model. We selected the Wells-Riley model because of a number of clear and compelling traits. First, data generated by the Wells-Riley model agree with observed data in airborne infection with measles and tuberculosis (*4**,**5*). Neither the Druett model nor other models of airborne infection with more specified variables and more complex mathematical forms (*32**,**33*) have been validated against epidemiologic data of any airborne infection. Second, the Wells-Riley model includes variables for environmental conditions and aerosol production rates not accounted for in the Druett model. Alternative models might allow for a sigmoidal growth in the probability for infection, i.e., the probability of infection increases monotonically; however, the rate of change first increases to a maximum and then decreases steadily to zero. While such a model is biologically plausible, experimental data to justify such a model are lacking. No models have been validated for inhalation anthrax in humans, which differs markedly from pulmonary tuberculosis and measles in its pathogenesis.

One possible approach to extending the Wells-Riley model in the future would be adding a susceptibility factor, y. With the current model formulation, as q increases, airborne infection approaches 100% certainty, i.e., the probability approaches 1. Increases in the other parameters (I, t, p and 1/Q) have a similar effect on the probability for airborne infection. An alternative model might be given by *C/S = y[1-exp(-Iqtp/Q)]* where 0 < y < 1. With such a model, as any of the parameters I, q, t, p, or 1/Q increase, the probability of airborne infection approaches 1. The factor y may be conceived as a fixed or random effect, or one that is conditional on measurable, individual-level susceptibility factors such as age or coexisting conditions. Additional data on susceptibility are needed, however, before such a parameter can be included with confidence.

Modeling depends on the assumptions used; the most critical of our assumptions is that all affected persons were exposed to similar quanta of *B. anthracis* from contaminated mail. More likely, the number of quanta decreased with "postal distance" from the index letters. Webb and Blaser allowed for this possibility by modeling the cross-contamination of letters resulting from contact with index letters passing through a prototypical postal system (*25*). Their model, however, did not allow for the environmental considerations that can mediate between aerosolization and infection. The Wells-Riley model allows for environmental considerations, and it can be adapted to variable exposure scenarios by stratifying by persons sharing a common quantum of exposure. To do that would require further modeling assumptions in the fashion of those made by Webb and Blaser. Epidemiologic data will likely never become available to test the validity of any of these models, but this modeling exercise demonstrates how the risk for infection is sensitive not only to the infectious dose but also to environmental parameters.

## Conclusion

The risk for airborne infection with *B. anthracis* is determined not only by the virulence of the organism but also by the balance between infectious aerosol production and removal, pulmonary ventilation rate, duration of exposure, and host susceptibility factors. Dilution ventilation of the indoor environment is an important determinant of the risk for infection. Enhanced room ventilation, UV germicidal irradiation, and other engineering control measures may be used to decrease the risk for infection. Although much research focuses on bacillary factors to improve our understanding of the pathogenesis of inhalation anthrax, our modeling data emphasize the need to better understand the complex interactions among host susceptibility factors, environmental factors, transmission mechanisms, and dose-response relationships in determining the risk of airborne infection with *B. anthracis* and other agents of bioterrorism.
